# The miR-196b miRNA inhibits the GATA6 intestinal transcription factor and is upregulated in colon cancer patients

**DOI:** 10.18632/oncotarget.13580

**Published:** 2016-11-25

**Authors:** Sebastian Fantini, Valentina Salsi, Luca Reggiani, Antonino Maiorana, Vincenzo Zappavigna

**Affiliations:** ^1^ Department of Life Sciences, Modena 41125, Italy; ^2^ Dipartimento di Scienze Mediche e Chirurgiche Materno-Infantili e dell'Adulto, University of Modena and Reggio Emilia, Modena 41125, Italy; ^3^ Dipartimento di Medicina Diagnostica, Clinica e di Sanità Pubblica, University of Modena and Reggio Emilia, Modena 41124, Italy

**Keywords:** colorectal cancer, gene regulation, gene expression, molecular mechanisms, molecular carcinogenesis

## Abstract

**Objective:**

To explore the possible misexpression of the microRNA *miR-196b* in colorectal cancer (CRC) and its role in controlling the expression of GATA6, a putative target gene crucial to intestinal cell homeostasis and tumorigenesis.

**Design:**

The expression of *miR-196b* was analysed by qRT-PCR in surgical resection samples from a cohort of sporadic colon cancer patients. Manipulations of *miR-196b* expression were performed to demonstrate its inhibition of GATA6 protein levels.

**Results:**

We found that *miR-196b* is significantly upregulated in pre-treatment surgical resection samples from a cohort of sporadic colon cancer patients. The upregulation of *miR-196b* correlates with less severe clinicopathological characteristics, such as early tumor stage and absence of lymph node metastases. We show that in CRC cells, *miR-196b* targets the mRNA of GATA6, a transcription factor involved in the homeostasis and differentiation of intestinal epithelial cells, and a positive regulator of the Wnt/β-catenin pathway. We moreover found that the increase of *miR-196b* correlates with a reduced GATA6 protein expression in colon cancer patients.

**Conclusion:**

Our results establish *miR-196b* as a post-transcriptional inhibitor of GATA6 in CRC cells, implicating *miR-196b* function in gene regulatory pathways crucial to intestinal cell homeostasis and tumorigenesis. Our results furthermore suggest a role of *miR-196b* expression in CRC, as an antagonist of GATA6 function in tumor cells, thus providing the basis for a potential targeting strategy for the treatment of CRC.

## INTRODUCTION

MicroRNAs (miRNAs) are involved in the regulation of a wide array of biological processes, ranging from cell cycle regulation to differentiation and development. Accordingly, the deregulation of miRNA expression has been implicated in several human diseases, including cancer [1]. While miRNA misregulation is recurrent in human cancer, for many malignancies it is still unknown whether the altered expression of miRNAs affects gene regulatory networks involved in the pathogenesis, and in this case, whether it is causative, correlative, or protective. miRNAs are small non-coding RNAs of ~20-24 nucleotides (nt) in length, which negatively control gene expression at the post-transcriptional level. They derive from larger primary miRNAs (pri-miRNAs), in many cases non-coding, which are processed by endonucleases to nuclear precursors (pre-miRNAs), and eventually to their mature cytoplasmic form. miRNAs typically bind to the 3′ UTR of their target mRNAs, and either inhibit translation or induce mRNA degradation [2, 3].

*miR-196* family members have been reported to participate in relevant biological processes, such as embryonic development (reviewed in 4) and tumorigenesis, where they have been found to be aberrantly expressed in various malignancies, including glioblastoma, colorectal cancer, melanoma, and leukemia (reviewed in 5). We recently reported the characterization of the *miR-196b* promoter. *miR-196b* is one of the three members of the evolutionarily conserved *miR-196* gene family, which includes in addition *miR-196a-1*, *miR-196a-2*, all located within the *HOX* gene clusters [6]. We reported that the evolutionarily conserved genomic region upstream to the *pri-miR-196b* transcriptional start site (TSS) contains several binding sites for Cdx and 5′Hox transcription factors, that these sites are bound in vivo by Cdx2, and that Cdx2 is necessary for the expression of *miR-196* in human embryonal carcinoma (EC) cells, establishing Cdx2 as a major regulator of *miR-196b* expression [7]. The *Caudal*-related homeobox transcription factor 2 (*CDX2*) gene is crucial for the normal development of the intestinal tract and for intestinal epithelium homeostasis [8–10]. In adult human, *CDX2* expression is confined to the small intestine and colon [11], and in colon cancer it has been proposed as a tumor suppressor gene [12–14]. Colorectal cancer (CRC) is worldwide the third most common cancer and the second major cause of cancer-related death [15]. Evidence has been increasing in the past years that CRC is a heterogeneous disease, whose molecular features decide the response to treatment and hence the prognosis [16].

Given the regulation of *miR-196b* by CDX2, a transcription factor important to intestinal epithelial cell homeostasis and disease, in this work, we wanted to explore the possible misregulation of *miR-196b* in colorectal cancer. We moreover wanted to verify whether *miR-196b* would control the expression of GATA6, a transcription factor involved in intestinal epithelial cell proliferation and CRC, which was predicted *in silico* as a target gene for *miR-196b*. *GATA6* codes for a member of the GATA family of transcription factors. GATA transcription factors play relevant roles in the development, proliferation, and differentiation of several organs [17, 18], of these GATA4, 5 and 6 are expressed in many tissues including the gastrointestinal tract [19]. More specifically, the *GATA6* gene is expressed in all the gastrointestinal epithelium with a peak of expression in the proliferative compartment of the crypts [20–22]. In accordance, the targeted inactivation of *GATA6* in mice causes early lethality due to the absence of endoderm differentiation [23, 24]. *GATA6* has been shown to be misregulated in colon cancer cells, suggesting a relevant role for GATA6 in the onset and progression of colon cancer [25, 26]. Indeed, GATA6 has been found to be a key player in a regulatory network converging into a pathway crucial to colorectal tumorigenesis, the Wnt/β-catenin signalling pathway [27].

Our results establish GATA6 as a target of *miR-196b* in colon cancer cells. We furthermore show that *miR-196b* is significantly upregulated in a cohort of sporadic colon cancer patients. Its upregulation was found to correlate with early stages of disease progression and with a marked reduction in GATA6 expression in colon cancer samples. Our results point to a regulatory mechanism, which could represent a potential target for the therapy of CRC.

## RESULTS

### *miR-196b* expression is upregulated in human colon cancer

As *miR-196b* was found [7] to be directly activated by the CDX2 intestinal-specific transcription factor [11], implicated in colon cancer pathogenesis [12–14], we sought to determine whether *miR-196b* expression was misregulated in colorectal cancer (CRC). To this end, we analysed the expression of *miR-196b* in intestinal tissue samples from a cohort of 63 colon cancer patients (see Table 1). The expression of *miR-196b* was determined by qRT-PCR on total RNA extracted from pre-treatment surgical resection samples of tumor mass and corresponding adjacent normal mucosa.

**Table 1:** Clinicopathological parameters of colon cancer patients

Clinical parameter	N. of patients (*n*=63)
Tumor Grade	
Grade 1	18
Grade 3	45
TNM Stage	
I - II	35
III - IV	28
Tumor Depth	
T 1-2	13
T 3-4	50
Lymph Node metastatisation	
N0	36
N+	27
Tumor Location	
Left*	37
Right**	26
Gender	
Male	35
Female	28

* = Descending + sigmoid

** = Ascending + transverse

**Figure 1:**
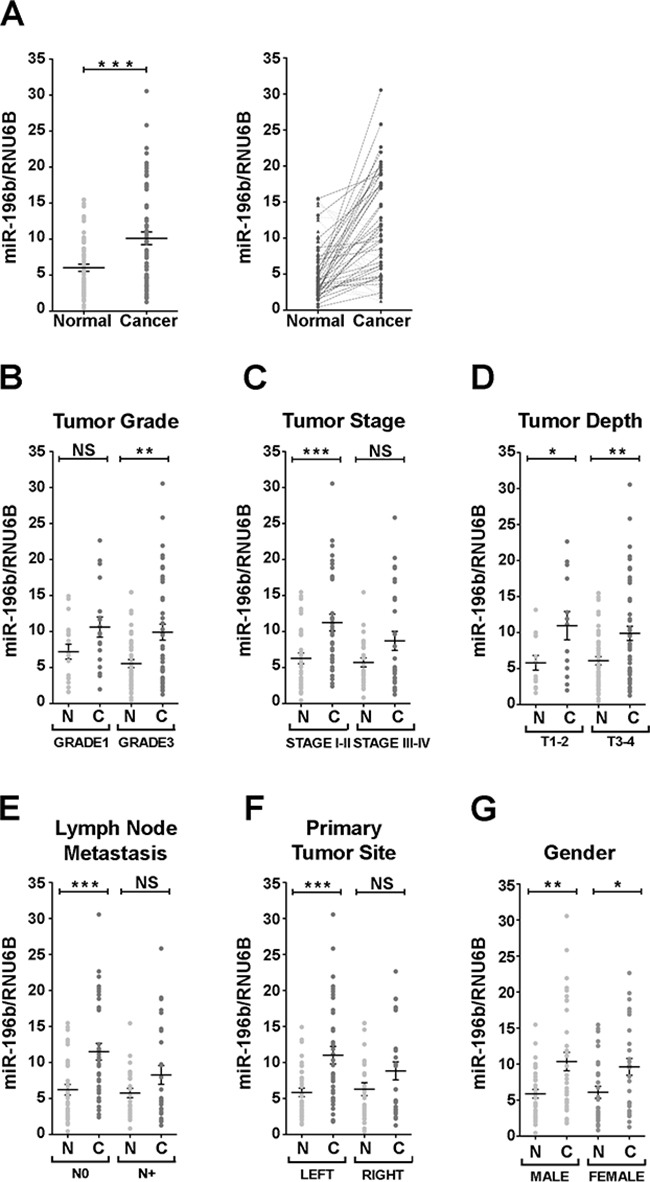
Expression of miR-196 in colon cancer human samples **A**. Relative expression of miR-196b vs RNU6B in matched healthy mucosae (light grey) and their corresponding primary colon cancer samples (dark grey) in aligned dot plot (*left panel*) and before-after (*right panel*) charts. In before-after graphs, patients with upregulated miR-196b expression in cancer lesions are indicated with circles and connected with a dark dashed line. Patients' samples with downregulated miR-196b are represented with triangles and connected by light dashed line. **B-G**. Aligned dot plot charts of patients classified according to the following tumor parameters: grade (B), stage (C), depth (D), lymph node metastasis (E), site of primary tumor (F) and gender (G). Bold horizontal bars represent mean expression level ± SEM. N = healthy mucosa. C = primary cancer. NS = not significant. * = p<0,05. ** = p<0,01. *** = p<0,001; paired t-test.

The expression of *miR-196b* was found to be significantly upregulated, with respect to paired normal adjacent tissue, in 40/63 (63.5%) of the colon cancer samples (Figure 1A). We then sought to correlate the misexpression of *miR-196b* in colon cancer samples with the clinicopathological characteristics of our cohort of patients. The misexpression of *miR-196b* was significantly associated to tumor grade. Grade 3 tumor samples showed an increase in *miR-196b* expression with respect to paired normal adjacent tissue, while grade 1 samples did not display a significant *miR-196b* upregulation (Figure 1B). *miR-196b* misexpression was moreover found to correlate with tumor stage, as the majority of colon cancer samples showing *miR-196b* upregulation belonged to the group of stage I and II tumors with respect to samples of the stage III and IV group (Figure 1C). A correlation was found in addition regarding lymph node metastases. *miR-196b* upregulation was significantly associated with the absence of lymph node invasion (N0) (Figure 1E). Finally, *miR-196b* misexpression also correlated with the site of origin of the primary tumor. The majority of samples showing *miR-196b* upregulation were derived from left colon (Figure 1F). No significant associations were found with the depth of tumor invasion (T) (Figure 1D) and with gender (Figure 1G), as well as with the diameter of the tumor mass and the age of the patients (Supplementary Figure S1)

Thus, collectively, our data show that *miR-196b* expression is significantly upregulated in colon cancer and that *miR-196b* upregulation is associated to several relevant clinicopathological features, such as tumor grade, stage and lymph node invasivity, suggesting that *miR-196b* misexpression may play a role in CRC pathogenesis by modulating the expression of target genes relevant to intestinal epithelial cell proliferation and/or differentiation.

### The GATA6 transcription factor is a target of *miR-196b*

**Figure 2:**
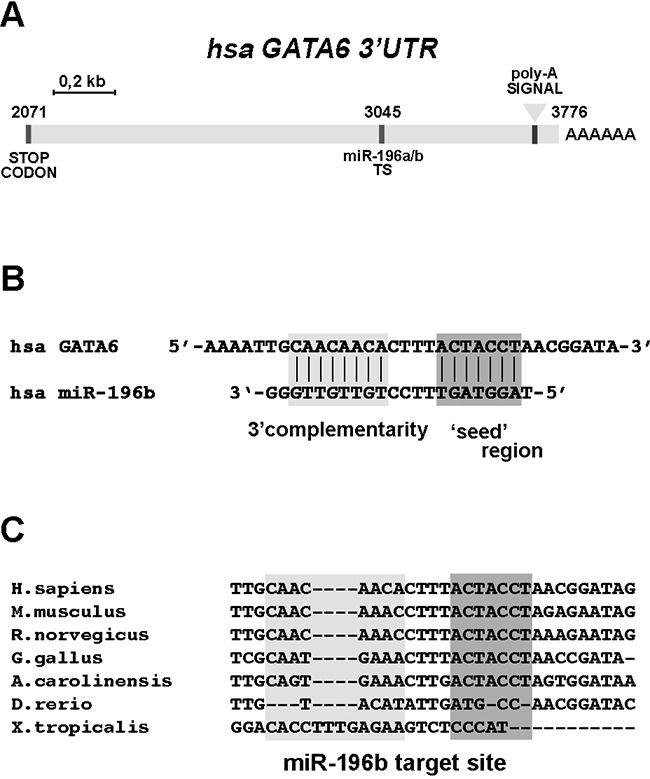
The 3′UTR sequence of GATA6 contains a conserved miR-196b target site **A**. Schematic representation of the human GATA6 3′ untranslated (3′UTR) region. Sequence positions are indicated, considering ‘+1’ the transcriptional start site (TSS) of the GATA6 transcript. TS, miR-196b target site. **B**. Interaction between miR-196b and the target site annotated on the 3′UTR of GATA6, as predicted by Targetscan software. **C**. Sequence comparison of the miR-196b target site. Dark grey boxes indicate the interaction mediated by the seed-region whereas the light rectangle indicates an additional pairing site driven by the 3′ half of miR-196b. The indicated species are: *Homo sapiens*, *Mus musculus*, *Rattus norvegicus*, *Gallus gallus*, *Anolis caroliniensis*, *Danio rerio*, *Xenopus tropicalis*.

We then searched for potential targets for the regulation by *miR-196b* that would be relevant to intestinal development and/or to CRC. Using the Targetscan, miRDB, PicTar, and DIANA-TarBase (see Materials and Methods) search platforms we identified the *GATA6* gene as a candidate *miR-196b* target gene. *GATA6* is a member of the GATA family of transcription factors, which play relevant roles in the development and differentiation of several organs, including the gastrointestinal tract [19]. The 3′ untranslated region (3′UTR) of the *GATA6* mRNA contains a single target sequence for *miR-196b*, 5′-TGCAACAACACTTTACTACCTA-3′, located approximately at the center of the 3′UTR (Figure 2A). The *GATA6 miR-196b* target sequence showed an 8mer seed match with a context++ score percentile of 99 and a total context++ score of -0.60 (Targetscan), and the target sequence was found to be evolutionarily highly conserved among land vertebrates (Figure 2C). We thus sought to confirm whether GATA6 is indeed negatively regulated by *miR-196b*. To this end we employed a luciferase reporter assay.

**Figure 3:**
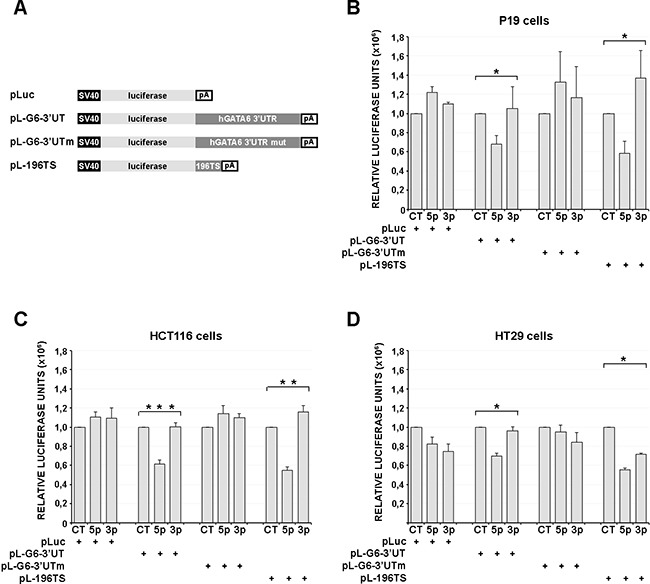
miR-196b functionally interacts with the GATA6 3′UTR **A**. Schematic representation of the reporter constructs used in luciferase transfection assays. SV40, SV40 early promoter. pA, SV40 late poly-adenylation signal. Luciferase activities assayed in cell extracts of P19 murine cells **B**., HCT116 and HT29 human CRC cells (**C** and **D**, respectively), transiently transfected with 200ng of the reporter constructs listed in (A) together with the synthetic microRNAs (40nM) indicated below. Bars represent the mean luciferase activity ± SEM of at least three independent experiments. * = p<0,05. ** = p<0,01. *** = p<0,001; non-parametric Kruskal-Wallis test followed by the Dunn's post-hoc test. CT, scrambled unrelated oligonucleotide. 5p, miR-196b-5p; 3p, miR-196b-3p

Three different luciferase reporter constructs were generated (Figure 3A), one containing the wild type GATA 3′ UTR region (pL-G6-3′UT), ranging approximately from the GATA6 stop codon to the GATA6 mRNA polyadenylation site (see Figure 2A), another containing the same GATA 3′ UTR region, but carrying a mutation of the *miR-196b* target sequence (pL-G6-3′UTm, Figure 3A), and a third one containing one copy of the *Hoxb8 miR-196b* target sequence as a positive control [28] (pL-196TS, Figure 3A). These reporter constructs were transfected, together with the mature *miR-196b* (miR-196b-5p, Ambion), with a control unrelated miRNA (Ambion), or with the control miR-196b-3p opposite strand *miR-196b*, in P19 murine embryonal carcinoma cells, or in HCT116 and HT29 human colon cancer cells. In P19 cells, which express very low levels of endogenous *miR-196b* and GATA6 (Supplementary Figure S2), the exogenous administration of mature *miR-196b* (miR-196b-5p) caused a significant decrease of the activity of the pL-G6-3′UT reporter, to ~68% with respect to pL-G6-3′UT cotransfected with the control miRNA, and to ~64% with respect to pL-G6-3′UT cotransfected miRNA processed from the opposite strand of the miR-196 precursor (miR-196b-3p) (Figure 3B). A comparable reduction was observed with the pL-196TS positive control (Figure 3B). Similarly, in HCT116 colon cancer cells, the activity of the pL-G6-3′UT and the pL-196TS reporters was significantly reduced by cotransfection of miR-196b-5p, to ~60% with respect to pL-G6-3′UT or pL-196TS cotransfected with the control miRNA and complementary miRNA (miR-196b-3p) (Figure 3C). Also in HT29 colon cancer cells miR-196b-5p transfection led to a decrease of pL-G6-3′UT and pL-196TS reporter activity, albeit to a lesser extent with respect to HCT116 cells, probably because of the higher expression levels of endogenous *miR-196b* and GATA6 (see Supplementary Figure S2) (Figure 3D). No, or only minor reductions of the reporter activities were observed with the control and miR-196b-3p miRNAs and with the mutated pL-G6-3′UTm reporter (Figure 3D).

Taken together, these results show that the GATA6 3′UTR can be targeted by *miR-196b* both in murine embryonal carcinoma as well as in human colon cancer cells.

### miR-196b exogenous expression decreases endogenous GATA6 protein levels in colon cancer cells

**Figure 4:**
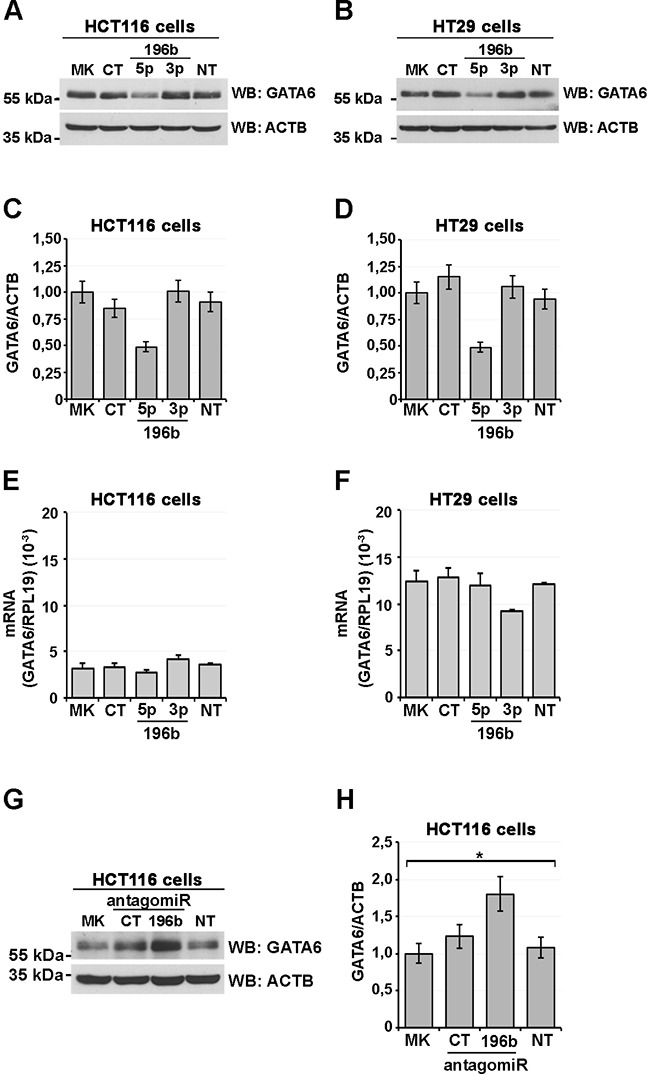
miR-196b expression reduces GATA6 protein levels Immunoblot analysis showing GATA6 levels in HCT116 (**A**) and HT29 (**B**) human CRC cells transfected with the indicated mature synthetic microRNAs. CT, scrambled oligonucleotide. 196b-5p, miR-196b-5p; 196b-3p, miR-196b-3p; MK, mock-transfected; NT, non transfected control. β-actin was used as loading control. Scanning densitometry of immunoblottings of HCT116 and HT29 cells (**C**, **D**, respectively). GATA6 mRNA levels assayed by qRT-PCR in total RNA from transfected HCT116 (**E**) and HT29 (**F**) cell lines. Expression levels were calculated according to the ΔΔCt method using the ribosomal protein L19 (RPL19) mRNA as a reference control. Immunoblot analysis showing GATA6 levels in HCT116. (**G**) HCT116 cells transfected with the microRNA antagomirs indicated below. CT, scrambled antagomir; Anti-196b, miR-196b-5p antagomir. Bars represent the mean ± SEM of at least three independent experiments.

We next wanted to further substantiate the regulation of GATA6 by *miR-196b* by verifying whether *miR-196b* overexpression would cause a decrease in cellular GATA6 protein. We thus transfected HCT116 and HT29 colon cancer cells, which express endogenous GATA6 (Supplementary Figure S2B), with the mature *miR-196b* (miR-196b-5p, Ambion), with a control, unrelated miRNA (Ambion), or with the control, opposite strand miR-196b-3p in HCT116 cells. miR-196b-5p overexpression caused a substantial decrease of endogenous GATA6 protein as revealed by immunoblotting (Figures 4A and 4C). Conversely, no changes in cellular GATA6 amounts were observed in mock-transfected cells, and in cells transfected with the control and miR-196b-3p miRNAs (Figures 4A and 4C). Similarly, in HT29 cells, GATA6 protein expression was significantly reduced only by cotransfection of miR-196b-5p, and not by cotransfection of the control and miR-196b-3p miRNAs (Figures 4B and 4D). In the same experiments, no significant changes in mRNA levels of GATA6 were observed both in HCT116 (Figure 4E) and HT29 (Figure 4F) colon cancer cells.

We then tested, using an anti-*miR-196b* antagomir, whether, reciprocally, a reduction of endogenous *miR-196b* would cause an increase in GATA6 protein levels. HCT116 cells, transfected with the anti-*miR-196b* antagomir showed a ~80% increase in GATA6 protein with respect to non-transfected or mock-transfected cells (Figure 4G-4H). Together, these results establish GATA6 as a target of *miR-196b* in colon cancer cells.

To test whether *miR-196b* exogenous expression would affect the proliferation and the cell cycle of colon cancer cells, HCT116 cells were transfected with the mature *miR-196b* (miR-196b-5p, Ambion), with a control, unrelated miRNA (Ambion), and cells were counted at different time points to measure the increase in vital cell numbers. Total protein extracts were also prepared to analyse phospho-Histone H3(Ser10) content by immunoblotting. Interestingly, the exogenous, transient expression of *miR-196b*, despite a detectable reduction in GATA6 protein levels, led to a slight, but reproducible increase in the cell growth rate and an initial increment in phospho-H3(Ser10) with respect to the exogenous expression of a control, unrelated miRNA (Supplementary Figure S3). However, at later timepoints, the fraction of mitotic cells, as revealed by phospho-H3(Ser10) amounts, decreased concomitantly with the decrease of GATA6 levels (Supplementary Figure S3C).

The cycle of HCT116 cells expressing exogenously *miR-196b* was also analysed, using monovariate and bivariate flow cytometry (Supplementary Figure S4). The expression of *miR-196b* caused an increase in the fraction of HCT116 cells in G2/M phase with respect to cells transfected with the control miRNA at 120 hrs after *miR-196b* transfection (Supplementary Figure S4A). No significant effects on the HCT116 cell cycle were observed in cells transfected with a control, unrelated miRNA (Supplementary Figure S4A). Also no significant changes in the fraction of apoptotic cells were observed (Supplementary Figure S4A). Bivariate analysis of DNA content and BrdU uptake showed, at 120 hrs after *miR-196b* transfection, an increase in the fraction of cells in late S phase with respect to cells transfected with a control, unrelated miRNA (Supplementary Figure S4B), suggesting a delay in the progression towards G2/M phase. Together, these results suggest that *miR-196b* exogenous expression alters both endogenous GATA6 protein levels and the proliferation and cell cycle of HCT116 cells.

### miR-196b expression increases upon colon cell differentiation concomitantly with a decrease of GATA6 protein levels

In an attempt to clarify the possible role(s) of *miR-196b* expression, and of its control over GATA6 protein levels, in colon cell physiology, we investigated whether variations of the expression of *miR-196b* would correlate with the differentiation of intestinal epithelial cells. To this end, we used Caco-2 cells, a human colon adenocarcinoma line, which undergoes spontaneous differentiation in culture displaying numerous differentiation hallmarks of intestinal epithelial cells [29, 30], and hence appeared to us as a profitable cell model to study colon cell differentiation in culture.

**Figure 5:**
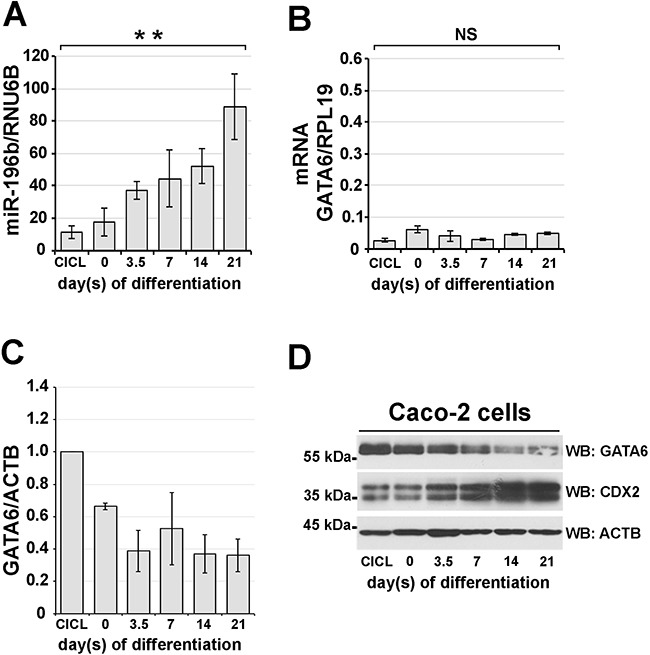
miR-196B and GATA6 expression levels inversely correlate during *in vitro* intestinal differentiation Caco-2 adenocarcinoma cells were induced to differentiate towards an enterocyte identity for 21 days upon confluency. miR-196b **A**. and GATA6 mRNA **B**. expression levels assayed by qRT-PCR in total RNA from differentiating Caco-2 cells. Expression levels were calculated according to the ΔΔCt method using RNU6B and RPL19, respectively, as reference genes. Scanning densitometry **C**. and immunoblotting **D**. of GATA6 protein during Caco-2 differentiation. Protein levels were normalized against β-actin expression. CDX2 protein was used as a marker of *in vitro* intestinal differentiation. CICL, cycling cells. ACTB, beta-Actin. Bars represent the mean ± SEM of at least three independent experiments. ** = p<0,01; one-way ANOVA followed by the Bonferroni's multiple comparison test.

Caco-2 cells were seeded at low density and cultured for three weeks; after reaching confluency they were collected at different time points to prepare nuclear extracts. Cycling Caco-2 cells displayed high levels of GATA6 protein as determined by immunoblotting (Figure 5D, see also Supplementary Figure S2B), which correlated with low levels of *miR-196b* expression (Figure 5A, see also Supplementary Figure S2A). During the first 7 days of differentiation GATA6 levels gradually declined to reach ~52% of the amount observed in cycling cells (Figure 5C-5D). In the following 14 days GATA6 levels continued to decline, reaching after 21 days their minimum, corresponding to ~36% of the amount observed in cycling cells (Figure 5C-5D). In parallel, *miR-196b* expression showed an 8-fold increase throughout the culturing time, reaching its maximal level at 21 days of culture (Figure 5A). We also verified possible variations in *GATA6* mRNA levels by qRT-PCR during the differentiation of Caco-2 cells, and observed no significant changes in *GATA6* transcript amounts, even after 21 days of culture (Figure 5B).

Thus, these data show that the steady increase of *miR-196b* expression corresponds to a steady decrease of GATA6 protein levels, which is not paralleled by a decrease in *GATA6* mRNA levels, further supporting the idea that GATA6 expression is controlled post-transcriptionally by *miR-196b*. Of note, the increase of *miR-196b* expression accompanying Caco-2 cell differentiation also correlated with the concomitant increase of expression of CDX2, a transcription factor, which we previously identified as being a regulator of *miR-196b* transcription, and is a differentiation marker of intestinal cells [7].

### GATA6 protein levels are reduced in tissue samples from colon cancer patients showing miR-196b upregulation

**Figure 6:**
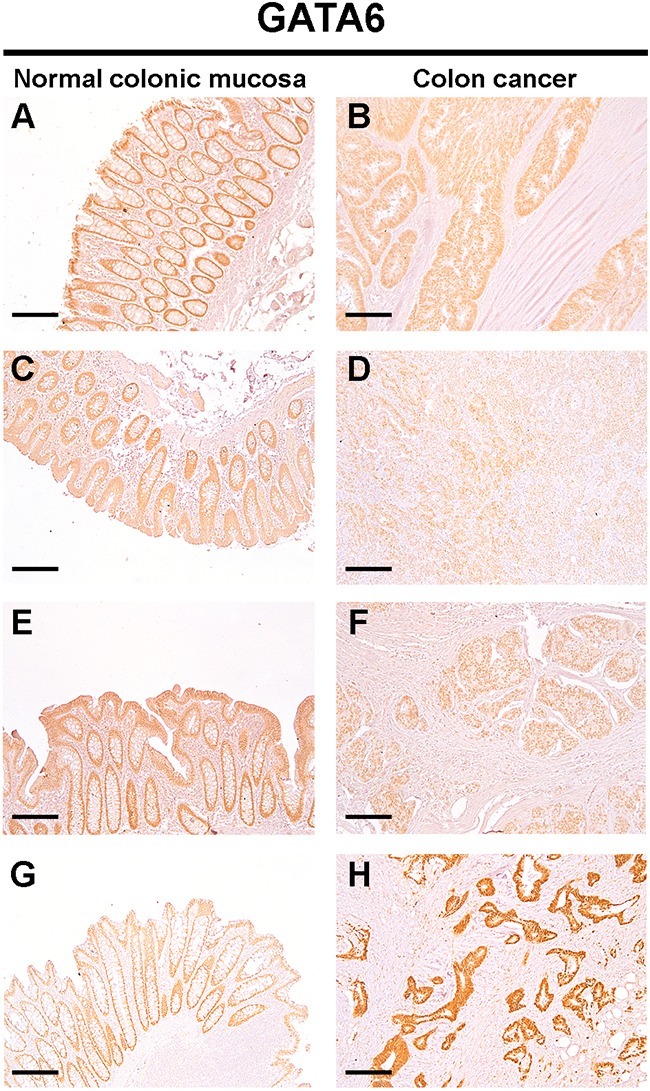
Protein levels of GATA6 are down-regulated in colon cancer tissues Expression of GATA6 in the normal colonic epithelium (**A**: **C**: **E**: **G**) and primary colon cancer (**B**: **D**: **F**: **H**) was detected by IHC. The positive staining was counterstained with hematoxylin. Images are representative of n=10 patients. Scale bar = 200 μm.

We next wanted to verify whether the upregulation of *miR-196b* expression correlated with a reduction in GATA6 expression in colon cancer tissue samples. To this end, we selected tissue samples showing *miR-196b* upregulation, and determined GATA6 protein expression by immunohistochemistry. Nine out of ten tissue samples (90%) displaying *miR-196b* upregulation showed a marked decrease in GATA6 protein levels in tumor tissue with respect to the adjacent normal colonic mucosa (representative results are shown in Figure 6, panels A to F). One sample having a *miR-196b* upregulation (~2-fold) displayed instead a more intense GATA6 expression in tumour tissue than in normal colonic mucosa (Figure 6, panels G and H).

These results confirm that *miR-196b* upregulation correlates with a reduction in GATA6 protein levels in colon cancer tissue samples.

## DISCUSSION

*miR-196* family members have been shown to participate in important biological processes, such as embryonic development [4] and tumorigenesis, where they have been found to be misexpressed in various malignancies [5]. We recently reported the characterization of the *miR-196b* promoter and established the CDX2 transcription factor as a major regulator of *miR-196b* expression [7]. The *CDX2* gene is crucial for the normal morphogenesis of the intestinal tract and for intestinal epithelium homeostasis [8–10], and in adult human its expression is confined to the small intestine and colon [11]. In colorectal cancer (CRC) *CDX2* it has been proposed as a tumor suppressor gene [12–14]. In this study, we therefore wanted to explore the possible involvement of *miR-196b* in CRC, by verifying at first whether it was misregulated in pre-treatment surgical resection samples from colon cancer patients. Our results show that *miR-196b* is indeed upregulated in the tumor mass of the majority of colon cancer samples analysed. We found in addition that the upregulation of *miR-196b* significantly correlates with several clinicopathological characteristics, such as tumor grade, stage, and lymph node metastases. Notably, *miR-196b* upregulation reveals in general to correlate with less severe clinicopathological characteristics, such as stage I/II of the disease and the absence of lymph node metastases, with the only possible exception of tumor grade, as *miR-196b* was found to be upregulated preferentially in grade 3 colon cancer samples.

In trying to understand which one(s) among the numerous predicted targets of *miR-196b* could play a role in the pathogenesis of CRC, our attention was attracted by the GATA6 transcription factor. Members of the GATA family of Zn-finger transcription factors play important roles in the control of cell proliferation and differentiation in several tissues and organs [17, 18]. GATA6, in particular, is expressed in the proliferating cells of the intestinal crypts and was shown to be required for crypt cell proliferation and migration [20, 21], and its targeted inactivation in mice causes early lethality due to a failure in endoderm differentiation [23, 24]. *GATA6* has been shown to be misexpressed in CRC cells from the earliest stages of dysplasia continuing to late metastatic lesions, pointing to a crucial role in the pathogenesis and progression of colon cancer [25, 26]. In accordance, in a nude mouse xenograft model, the tumorigenicity of CRC cells has been shown to be blocked by a knocking down GATA6 [27, 31].

Being GATA6 an in silico predicted target of *miR-196b*, we decided to verify whether it was indeed inhibited by *miR-196b* and whether *miR-196b* upregulation would reduce GATA6 protein levels in CRC cells. We found that a target sequence for *miR-196b*, located within the 3′UTR of the *GATA6* mRNA is necessary for mediating post-transcriptional regulation of GATA6 expression by *miR-196b*. In CRC cells, endogenous GATA6 protein levels were found to be downregulated by *miR-196b* overexpression, and in the Caco-2 intestinal epithelial cell differentiation model, the decrease in GATA6 protein expression during differentiation correlated with an upregulation of *miR-196b* expression. Our results thus establish GATA6 as a target of *miR-196b* post-transcriptional regulation in CRC cells, implicating *miR-196b* function in gene regulatory pathways crucial to intestinal cell homeostasis and tumorigenesis. GATA6 is in fact a positive regulator of a set of genes relevant to CRC carcinogenesis and progression, such as uPA, REG4 [26, 31], and most importantly LGR5 [27]. LGR5 is an R-spondin receptor, which has been identified as a marker of the intestinal crypt basal columnar stem cells [32]. LGR5 is a target gene of the canonical Wnt/β-catenin signalling pathway, and at the same time potentiates Wnt/β-catenin signalling by enhancing Wnt-induced LRP6 phosphorylation [33]. Thus GATA6, by controlling the transcription of LGR5, positively impacts on Wnt/β-catenin signalling in intestinal epithelial stem cells [27].

The vast majority of colorectal cancers (~90%) carry mutations in one of two genes involved in the canonical Wnt/β-catenin signalling pathway: the adenomatous polyposis coli (APC) and -catenin (CTNNB1) genes. More than 80% of colorectal cancers have mutations in the APC tumor suppressor gene. Mutation of the-catenin gene is mutually exclusive to APC mutation, and about half of all CRCs with wild-type APC have mutations in the CTNNB1 gene [34]. Experimental evidence has been accumulating that intestinal tumorigenesis is caused by the aberrant activation of Wnt/β-catenin signaling in epithelial stem cells [32]. It has been furthermore recently shown that the restoration of APC function causes established colorectal cancer to revert to normal epithelia, even in the presence of mutations in the Tp53 and Kras genes, demonstrating the dependency of CRC cells on constitutively active Wnt signaling [35].

The negative regulation of GATA6 expression by *miR-196b* likely impinges on Wnt/β-catenin signaling via the downregulation of LGR5 transcription. A similar control has been reported to be exerted by *miR-363*, which was found to target the *GATA6* mRNA and to cause a decrease in the tumorigenicity of CRC cells [27]. *miR-363*, however, unlike *miR-196b*, has been shown to be downregulated in CRC tissue samples [27]. We propose that the upregulation of *miR-196b*, which we observed in CRC tissue samples and found to correlate with a reduction of GATA6 protein levels, could represent a putative protective response of CRC cells to the aberrant activation of Wnt/β-catenin signaling. Indeed, *miR-196* family members, including *miR-196b*, have been reported to act as metastasis suppressors in breast cancer cells, being their enforced expression able to inhibit cell invasion *in vitro* and metastasis formation *in vivo* [36]. Interestingly, we found that the exogenous expression of *miR-196b* in HCT116 colon cancer cells, despite causing a detectable decrease in GATA6 protein levels, slightly increases cell proliferation and, initially, the fraction of mitotic cells. Mitotic cells, however, subsequently decrease concomitantly with the further reduction in GATA6 levels. The relatively moderate and delayed effect of *miR-196* exogenous expression could rest on the transient mode of expression of *miR-196* within cells. Alternatively, the mild effect of *miR-196* exogenous expression on HCT116 cell proliferation could rest on mechanisms other than GATA6 downregulation. Further work will be required to highlight the effect of *miR-196b* upregulation on LGR5 expression, on Wnt/β-catenin signaling, and on tumorigenicity in CRC cells. Nevertheless, the findings that GATA6 expression is significantly reduced in tissue samples showing upregulation of *miR-196b* and that *miR-196b* upregulation correlates with less severe clinicopathological features would suggest a possible protective role of *miR-196b* upregulation in CRC carcinogenesis.

## MATERIALS AND METHODS

### Patients

Formalin-fixed, paraffin-embedded (FFPE) primary cancer tissues and matched colonic mucosae from 63 colon cancer patients were analyzed. All cases were enrolled at the Policlinico Universitario of Modena hospital, Modena, Italy. Only patients affected by sporadic colon cancer samples were included in this study and patients treated with radio- or chemo-therapy prior to surgery together with patients affected by mucinous cancer were excluded. All the cases were collected between 2008 and 2014.

### Cell culture and cell cycle analyses

P19 cells (ATCC# CRL-1825) mouse embryonal carcinoma cells were cultured in Minimum Essential Medium Alpha (αMEM, GIBCO), with nucleosides. Caco-2 (ATCC# HTB-37) and HT29 (ATCC# HTB-38) human colorectal adenocarcinoma cells were cultured in Dulbecco's Modified Eagle Medium (DMEM, Euroclone). HCT116 (ATCC# CCL-247) human colorectal carcinoma cells were cultured in Iscove's Modified Dulbecco's Medium (IMDM, Euroclone). All the media were supplemented with 10% foetal bovine serum, 2mM L-glutamine, 100U/mL penicillin and 100 μg/mL streptomycin. For *in vitro* differentiation assays, Caco-2 cells were cultured for 21 days after they reached confluence [37]. Medium was replaced three times a week. Cell growth was assessed using the trypan blue dye exclusion staining and cell counting using a Burker chamber. Monovariate and bivariate cell cycle analyses were performed as described in [38, 39]. Briefly, DNA content was measured by propidium iodide (50mg/ml) staining of and cytofluorimetry. Cells were pulsed with 10mM bromodeoxyuridine (BrdU, Sigma-Aldrich) for 30min at 37°C and BrdU incorporation was detected by cytofluorimetry using an anti-BrdU antibody (Becton Dickinson).

### Transfection assays

For details on transfection experiments see Supplementary Materials and Methods.

### Plasmid constructs

For details on plasmid construction see Supplementary Materials and Methods.

### RNA extraction and qRT-PCR

Total RNA from cultured cells was isolated by TRIzol (Invitrogen) according to the manufacturer instructions. RNA from FFPE tissues (three 15μm-thick sections for each sample) was isolated using the RecoverAll isolation kit (Ambion) following the manufacturer's protocol.

Mature *miR-196b* and the endogenous control RNU6B were reverse transcribed and detected by real-time (qRT-PCR) with TaqMan® microRNA assays (Applied Biosystems) following the manufacturer's protocol. Synthesis of cDNA was done starting from 3 μg of total RNA using M-MLV reverse transcriptase (Promega), oligo(dT) and random primers. In all qRT-PCR experiments, the relative quantification was performed according to the ΔΔCt method [40]. Oligonucleotides used are listed in Supplementary Materials and Methods.

### Protein extracts and immunoblotting

For details on immunoblotting and protein extraction see Supplementary Materials and Methods.

### Immunostainings

Paraffin sections were rehydrated following ethanol series and antigen retrieval was performed in Tris-HCl 10mM, EDTA 1mM in pressure cooker at 121°C for 20min. Slides were bleached in 0,3% H_2_O_2_ and blocked in 2%BSA, 0,1%TWEEN-20. Sections were then incubated overnight with primary GATA6 antibody (1:100, AF1700, R&D System) at 4°C and for one hour with biotinylated anti-goat antibody (1:200) at room temperature, followed by 50min incubation with AB complex (Vector Vectastain Elite kit). Signal was visualized with diaminobenzidine and counterstained with Harris hematoxylin according to the standard procedure.

### Bioinformatics and statistical analyses

For details on statistics and bioinformatics see Supplementary Materials and Methods.

## SUPPLEMENTARY FIGURES



## References

[1] Calin GA, Croce CM (2006). MicroRNA signatures in human cancers. Nat Rev Cancer.

[2] Bartel DP (2004). MicroRNAs: genomics, biogenesis, mechanism, and function. Cell.

[3] Bartel DP (2009). MicroRNAs: target recognition and regulatory functions. Cell.

[4] Mansfield JH, Harfe BD, Nissen R, Obenauer J, Srineel J, Chaudhuri A, Farzan-Kashani R, Zuker M, Pasquinelli AE, Ruvkun G, Sharp PA, Tabin CJ, McManus MT (2004). MicroRNA-responsive (sensor’ transgenes uncover Hox-like and other developmentally regulated patterns of vertebrate microRNA expression. Nat Genet.

[5] Chen C, Zhang Y, Zhang L, Weakley SM, Yao Q (2011). MicroRNA-196: critical roles and clinical applications in development and cancer. J Cell Mol Med.

[6] Mansfield JH, McGlinn E (2012). Evolution, expression, and developmental function of Hox-embedded miRNAs. Curr Top Dev Biol.

[7] Fantini S, Salsi V, Vitobello A, Rijli FM, Zappavigna V (2015). MicroRNA-196b is transcribed from an autonomous promoter and is directly regulated by Cdx2 and by posterior Hox proteins during embryogenesis. Biochim Biophys Acta.

[8] Chawengsaksophak K, James R, Hammond VE, Kontgen F, Beck F (1997). Homeosis and intestinal tumours in Cdx2 mutant mice. Nature.

[9] Simmini S, Bialecka M, Huch M, Kester L, van de Wetering M, Sato T, Beck F, van Oudenaarden A, Clevers H, Deschamps J (2014). Transformation of intestinal stem cells into gastric stem cells on loss of transcription factor Cdx2. Nat Commun.

[10] Verzi MP, Shin H, Ho LL, Liu XS, Shivdasani RA (2011). Essential and redundant functions of caudal family proteins in activating adult intestinal genes. Mol Cell Biol.

[11] Suh E, Chen L, Taylor J, Traber PG (1994). A homeodomain protein related to caudal regulates intestine-specific gene transcription. Mol Cell Biol.

[12] Hryniuk A, Grainger S, Savory JG, Lohnes D (2014). Cdx1 and Cdx2 function as tumor suppressors. J Biol Chem.

[13] Aoki K, Tamai Y, Horiike S, Oshima M, Taketo MM (2003). Colonic polyposis caused by mTOR-mediated chromosomal instability in Apc+/Delta716 Cdx2+/− compound mutant mice. Nat Genet.

[14] Bonhomme C, Duluc I, Martin E, Chawengsaksophak K, Chenard MP, Kedinger M, Beck F, Freund JN, Domon-Dell C (2003). The Cdx2 homeobox gene has a tumour suppressor function in the distal colon in addition to a homeotic role during gut development. Gut.

[15] Jemal A, Bray F, Center MM, Ferlay J, Ward E, Forman D (2011). Global cancer statistics. CA Cancer J Clin.

[16] Sridharan M, Hubbard JM, Grothey A (2014). Colorectal cancer: how emerging molecular understanding affects treatment decisions. Oncology.

[17] Zheng R, Blobel GA (2010). GATA Transcription Factors and Cancer. Genes Cancer.

[18] Aronson BE, Stapleton KA, Krasinski SD (2014). Role of GATA factors in development, differentiation, and homeostasis of the small intestinal epithelium. Am J Physiol Gastrointest Liver Physiol.

[19] Molkentin JD (2000). The zinc finger-containing transcription factors GATA-4, -5, and -6. Ubiquitously expressed regulators of tissue-specific gene expression. J Biol Chem.

[20] Gao X, Sedgwick T, Shi YB, Evans T (1998). Distinct functions are implicated for the GATA-4, -5, and -6 transcription factors in the regulation of intestine epithelial cell differentiation. Mol Cell Biol.

[21] Beuling E, Aronson BE, Tran LM, Stapleton KA, ter Horst EN, Vissers LA, Verzi MP, Krasinski SD (2012). GATA6 is required for proliferation, migration, secretory cell maturation, and gene expression in the mature mouse colon. Mol Cell Biol.

[22] Beuling E, Baffour-Awuah NY, Stapleton KA, Aronson BE, Noah TK, Shroyer NF, Duncan SA, Fleet JC, Krasinski SD (2011). GATA factors regulate proliferation, differentiation, and gene expression in small intestine of mature mice. Gastroenterology.

[23] Morrisey EE, Tang Z, Sigrist K, Lu MM, Jiang F, Ip HS, Parmacek MS (1998). GATA6 regulates HNF4 and is required for differentiation of visceral endoderm in the mouse embryo. Genes Dev.

[24] Koutsourakis M, Langeveld A, Patient R, Beddington R, Grosveld F (1999). The transcription factor GATA6 is essential for early extraembryonic development. Development.

[25] Haveri H, Westerholm-Ormio M, Lindfors K, Maki M, Savilahti E, Andersson LC, Heikinheimo M (2008). Transcription factors GATA-4 and GATA-6 in normal and neoplastic human gastrointestinal mucosa. BMC Gastroenterol.

[26] Belaguli NS, Aftab M, Rigi M, Zhang M, Albo D, Berger DH (2010). GATA6 promotes colon cancer cell invasion by regulating urokinase plasminogen activator gene expression. Neoplasia.

[27] Tsuji S, Kawasaki Y, Furukawa S, Taniue K, Hayashi T, Okuno M, Hiyoshi M, Kitayama J, Akiyama T (2014). The miR-363-GATA6-Lgr5 pathway is critical for colorectal tumourigenesis. Nat Commun.

[28] Hornstein E, Mansfield JH, Yekta S, Hu JK, Harfe BD, McManus MT, Baskerville S, Bartel DP, Tabin CJ (2005). The microRNA miR-196 acts upstream of Hoxb8 and Shh in limb development. Nature.

[29] Saaf AM, Halbleib JM, Chen X, Yuen ST, Leung SY, Nelson WJ, Brown PO (2007). Parallels between global transcriptional programs of polarizing Caco-2 intestinal epithelial cells in vitro and gene expression programs in normal colon and colon cancer. Mol Biol Cell.

[30] Christensen J, El-Gebali S, Natoli M, Sengstag T, Delorenzi M, Bentz S, Bouzourene H, Rumbo M, Felsani A, Siissalo S, Hirvonen J, Vila MR, Saletti P, Aguet M, Anderle P (2012). Defining new criteria for selection of cell-based intestinal models using publicly available databases. BMC Genomics.

[31] Kawasaki Y, Matsumura K, Miyamoto M, Tsuji S, Okuno M, Suda S, Hiyoshi M, Kitayama J, Akiyama T (2015). REG4 is a transcriptional target of GATA6 and is essential for colorectal tumorigenesis. Sci Rep.

[32] Barker N, Ridgway RA, van Es JH, van de Wetering M, Begthel H, van den Born M, Danenberg E, Clarke AR, Sansom OJ, Clevers H (2009). Crypt stem cells as the cells-of-origin of intestinal cancer. Nature.

[33] Carmon KS, Gong X, Lin Q, Thomas A, Liu Q (2011). R-spondins function as ligands of the orphan receptors LGR4 and LGR5 to regulate Wnt/beta-catenin signaling. Proc Natl Acad Sci U S A.

[34] (2012). Cancer-Genome-Atlas-Network. Comprehensive molecular characterization of human colon and rectal cancer. Nature.

[35] Dow LE, O‗Rourke KP, Simon J, Tschaharganeh DF, van Es JH, Clevers H, Lowe SW (2015). Apc Restoration Promotes Cellular Differentiation and Reestablishes Crypt Homeostasis in Colorectal Cancer. Cell.

[36] Li Y, Zhang M, Chen H, Dong Z, Ganapathy V, Thangaraju M, Huang S (2010). Ratio of miR-196s to HOXC8 messenger RNA correlates with breast cancer cell migration and metastasis. Cancer Res.

[37] Pinto M, Robine-Leon S, Apay M, Keddinger M, Triadou N, Dussaulx E, Lacroix B, Simon-Assman P, Hafen K, Fogh J, Zweibaum A (1983). Enterocyte-like Differentiation and Polarization of the human Colon Carcinoma Cell Line Caco-2 in Culture. Biol Cell.

[38] Salsi V, Ferrari S, Gorello P, Fantini S, Chiavolelli F, Mecucci C, Zappavigna V (2014). NUP98 Fusion Oncoproteins Promote Aneuploidy by Attenuating the Mitotic Spindle Checkpoint. Cancer Res.

[39] Salsi V, Fantini S, Zappavigna V (2015). NUP98 fusion oncoproteins interact with the APC/C(Cdc20) as a pseudosubstrate and prevent mitotic checkpoint complex binding. Cell Cycle.

[40] Livak KJ, Schmittgen TD (2001). Analysis of relative gene expression data using real-time quantitative PCR and the 2(−Delta Delta C(T)) Method. Methods.

[41] Fantini S, Vaccari G, Brison N, Debeer P, Tylzanowski P, Zappavigna V (2009). A G220V substitution within the N-terminal transcription regulating domain of HOXD13 causes a variant synpolydactyly phenotype. Hum Mol Genet.

