# Supplementary material for: The miR-196b miRNA inhibits the GATA6 intestinal transcription factor and is upregulated in colon cancer patients
Source: Oncotarget. 2016 Nov 25;8(3):4747–59. doi: 10.18632/oncotarget.13580 (PMC5354868; doi:10.18632/oncotarget.13580)
Supplement: Supplementary file 1 [file oncotarget-08-4747-s001.pdf]

The miR-196b miRNA inhibits the GATA6 intestinal transcription factor and is upregulated in colon cancer patients

SUPPLEMENTARY MATERIALS AND METHODS

Plasmid constructs

A 1335bp fragment of the human GATA6 3'UTR was obtained by RT-PCR amplification from cDNA of HEK293 cells using the following primers: GATA6-3'UTR F: 5'-GCCTCACTCCACTCGTGTC-3'; GATA6-3'UTR R: 5'-CTGTCCCCAATGCACATG-3'. The amplified fragment was cloned and sequence-verified into the KpnI and XbaI sites of the pGL3-PROM-3'PLK reporter vector (kind gift of Dr. G. Marziali), a modified version of pGL3-PROM vector (Promega) with the poly-linker sequence downstream to the luciferase coding sequence, to obtain the pL-G6-3'UT construct. The pL-G6-3'UTm mutant was generated via splicing by overlapping extension (SOE), using the wild-type pL-G6-3'UT vector as template, and mutating the miR-196 target site using the following primers: GATA6-3'MUT F: 5'-CAACAACACTTTCAGCAAGCACGGATAGCATTTGTAAATAC-3' and GATA6-3'MUT R: 5'-AATGCTATCCGTGCTTGCTGAAAGTGTTGTTGCAATTTTTCC-3'. The mutant was cloned into the KpnI and XbaI site of the pGL3-PROM-3'PLK reporter vector and sequence-verified. Finally, the pL-196TS reporter construct was generated cloning the oligonucleotide 5'-CGGATCCGAGCTCCCAACAACATGAAACTGCCTATTCT-3' into the XbaI and KpnI site of the pGL3-PROM-3'PLK reporter vector.

Transfection assays

For transfection of microRNAs and antagomirs, cells were seeded $3x10^5$/6-well 24 hrs before the transfection. Synthetic mature microRNA (AM17100 Pre-miR-196b-5p and Pre-miR-196b-3p miRNA precursors, Ambion) and microRNA antagomirs (AM17000 anti-miR-196b-5p inhibitor, Ambion) were transfected at a final concentration of 100nM using Lipofectamine 2000 (Invitrogen) and collected 48 hrs after the transfection. Unrelated Pre-miR-CTRL#1 (AM17110) and Anti-miR-CTRL#1 (AM17010) were used as negative control. In reporter inhibition assays, cells were seeded $1x10^5$ in 24-well the day before the transfections. 200ng of reporter constructs were co-transfected with 25ng of pCMV-BGal (Clonetech) and 40nM of Pre-miR. Cells were lysed 24 hrs after the transfection and assayed for luciferase and b-Galactosidase as described in [41].

RNA extraction and qRT-PCR

Quantitative PCR was performed with the following oligonucleotides: hCDX2 F: 5′-GTGCTAAACCCCACCGTCAC-3′; hCDX2 R: 5′-CTGAGGAGTCTAGCAGAGTC-3′; hGATA6 F:5'-TCAAACCAGGAAACGAAAACC-3', hGATA6 R: 5'-TTGGAGTCATGGGAATGGAAT-3', hRPL19 F: 5'-GGGCATAGGTAAGCGGAAGG-3', hRPL19 R: 5'-TCAGGTACAGGCTGTGATACA-3'.

Protein extracts and immunoblotting

Whole cell extracts were prepared through repeated freeze and thaw cycles and extraction in 10 mM Hepes pH7.9, 300 mM NaCl, 0.1 mM EGTA, 5% glycerol, 0,5% Triton X-100 and proteases and phosphatase inhibitors. Immunoblotting analyses were performed with anti-CDX2 antibody (Abcam, ab76541); anti-GATA6 (AF1700, R&D System), anti-Actin (I-19; SC-1616) and anti-phospho-Histone H3 (Ser10) (#9701, Cell Signaling Technology). Protein expression in immunoblots was quantified using the NIH ImageJ software.

Cycling and differentiating Caco-2 cells were resuspended in Lysis Buffer (140mM NaCl, 1,5mM $MgCl_2$, 0,5% NP-40, 10mM Tris-HCl pH 8.6 and protease and phosphatase inhibitors) and stratified on a 24% sucrose cushion. The pelletted nuclei were resuspended in 20mM Tris-HCl pH 7.6, 420mM KCl, 0,2mM EDTA, 1,5mM MgCl2, 25% glycerol and protease and phosphatase inhibitors.

Bioinformatics and statistical analyses

To identify putative target genes of miR-196, bioinformatics analyses were performed using: TargetScan (http://www.targetscan.org/); miRDB (http://mirdb.org/miRDB/), DIANA-TarBase (http://diana.imis.athena-innovation.gr) and PicTar (http://pictar.mdc-berlin.de/). Statistical analyses were performed with GraphPad Prism version 6.0 software. For comparison of more than two independent groups, the Kruskal-Wallis followed by Dunn's post-hoc test was used for luciferase assay, the one-way ANOVA followed by the Bonferroni's multiple comparison test for qRT-PCR analyses. In patients' analysis, the paired t-test was used to evaluate

significant differences between two matched pair groups. Pearson's correlation coefficient (r) was used to measure correlation, and linear regression was used to calculate the slope of the best fit line. Outliers were identified and excluded from the analyses using the ROUT method by GraphPad Prism.

SUPPLEMENTARY FIGURES

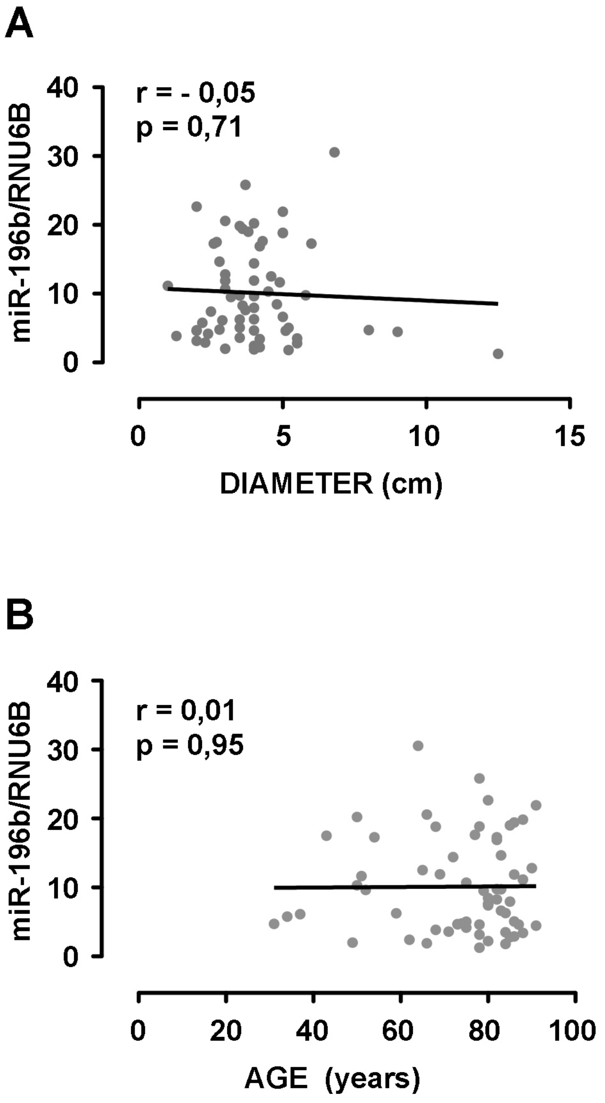

Supplementary Figure S1: miR-196b upregulation in CRC tissues is not correlated to tumor diameter and patients age. Pearson's correlation coefficient (r) was calculated between relative expression levels of miR-196b (miR-196b/RNU6B) in CRC tissues and diameter of the primary tumor (**A**) or age of patients at the time of surgery (**B**). Black lines represent linear regression lines.

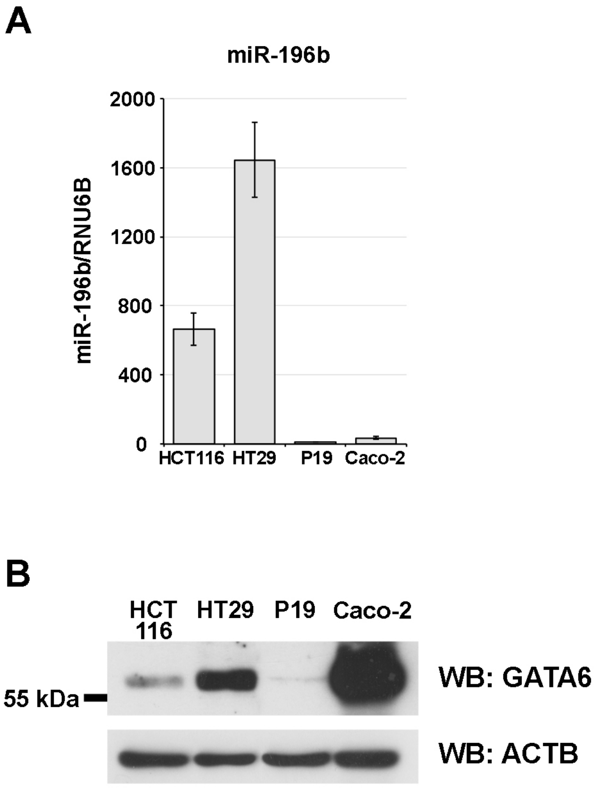

Supplementary Figure S2: miR-196b and GATA6 expression levels in human CRC cells. A. miR-196b expression levels assayed by qRT-PCR in total RNA from the cultured cell lines indicated below. Undifferentiated Caco2 cells were used for RNA extraction. Expression levels were calculated according to the ΔΔCt method using RNU6B as a reference gene. **B.** Immunoblotting of GATA6 expression in whole cell extracts prepared from the indicated cell lines. β-actin (ACTB) was used as loading control.

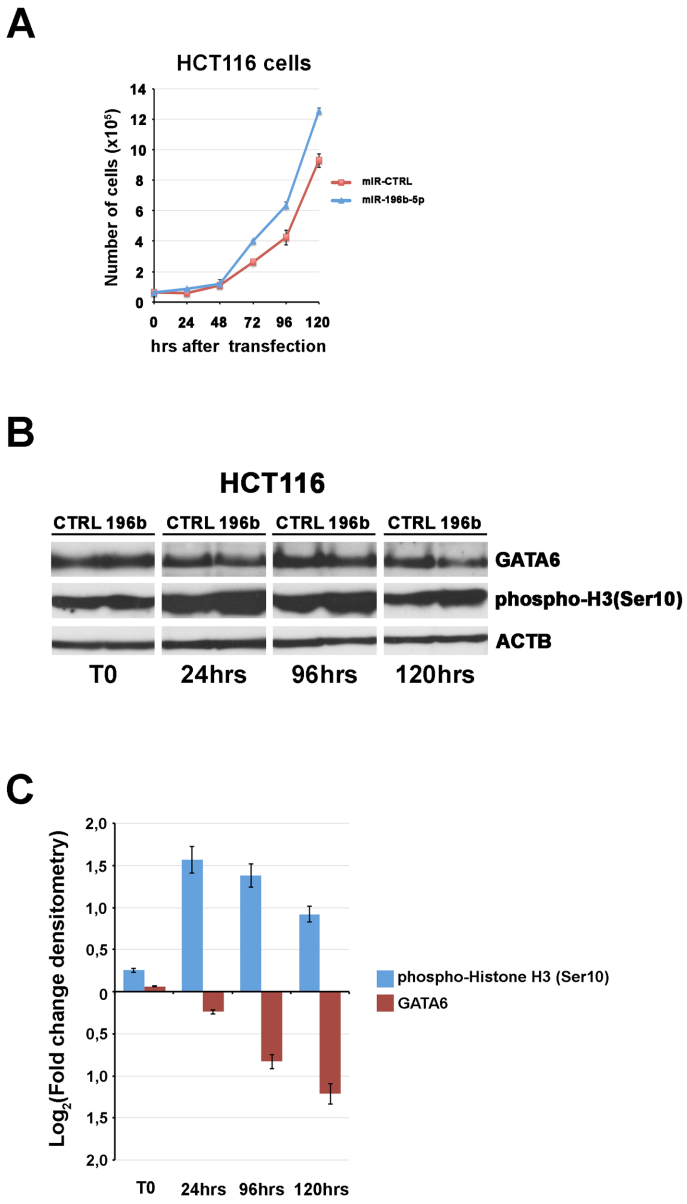

Supplementary Figure S3: miR-196b overexpression affects proliferation and mitotic index of HCT116 cells. A. Graph represents the number of vital cells (trypan blue staining) counted at different time points after the transfection of miR-196b-5p (blue) or of a control, unrelated miRNA (red). **B.** A representative example of an immunoblotting analysis, at the indicated time points, of GATA6 and phospho-Histone H3 (phospho-H3(Ser10)) in HCT116 cells transfected with an unrelated miRNA (CTRL) and miR-196b-5p (196b). β-actin (ACTB) was used as loading control. **C.** Scanning densitometry of immunoblottings of HCT116 transfected with unrelated miRNA and miR-196b-5p. Bars represent the mean Log_2 ± the SEM (N=3) of the fold change of phospho-Histone H3(Ser10)/ACTB (blue) and GATA6/ACTB (red) of cells transfected with miR-196b-5p vs control miRNA.

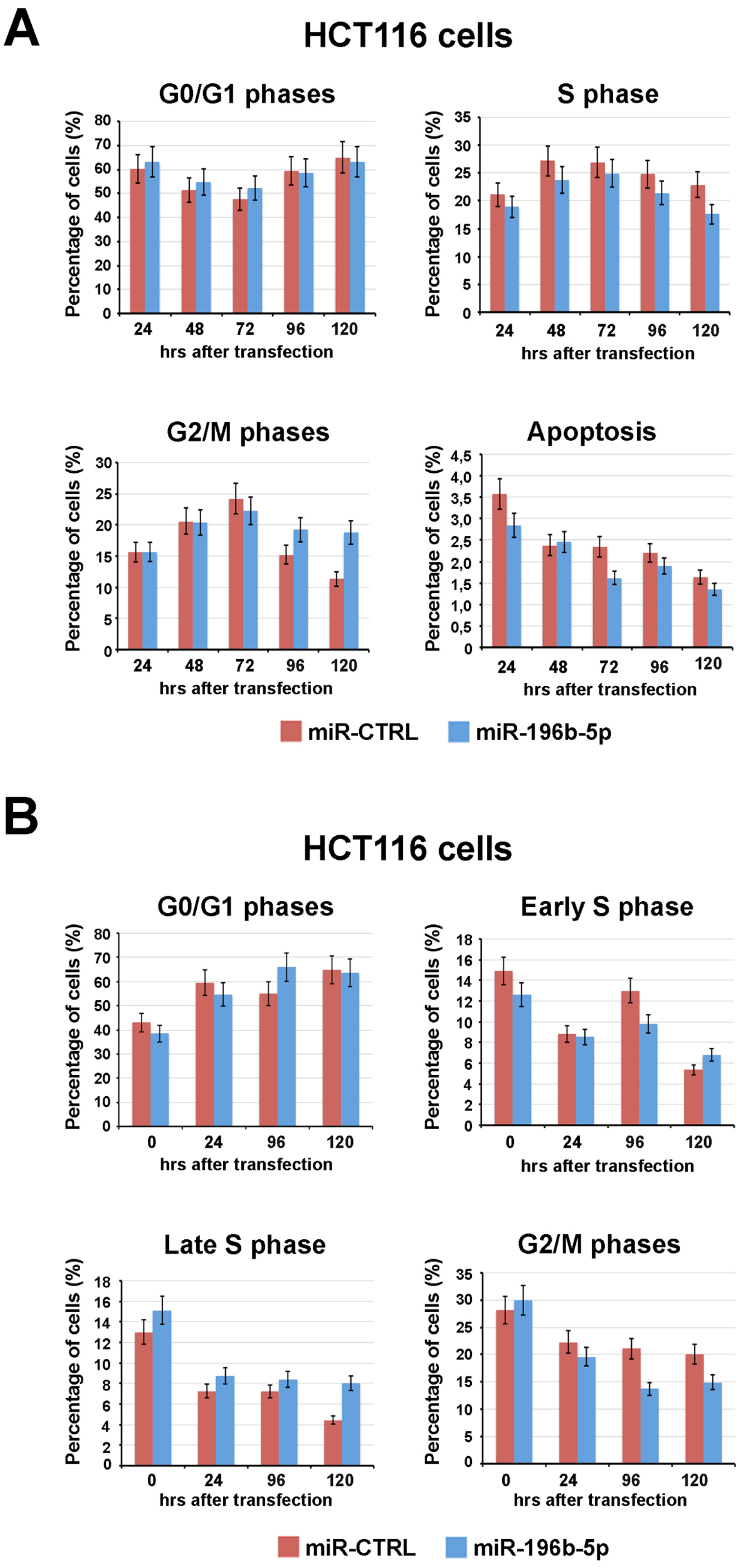

Supplementary Figure S4: miR-196b overexpression alters the HCT116 cell cycle. A. Flow cytometry of the DNA content (propidium iodide, PI) of HCT116 cells transiently transfected with mature miR-196b-5p (blue) or control unrelated miRNA (CTRL, red). **B.** BrdU/PI bivariate cytofluorimetric analysis to measure the replicative DNA content of HCT116 cells transfected with mature miR-196b-5p (blue) or control unrelated miRNA (CTRL, red). The chart represents the percentages of cells in the indicated cell cycle phases, at different time points after transfection. Bars represent the mean percentage ± the SEM of three independent experiments.

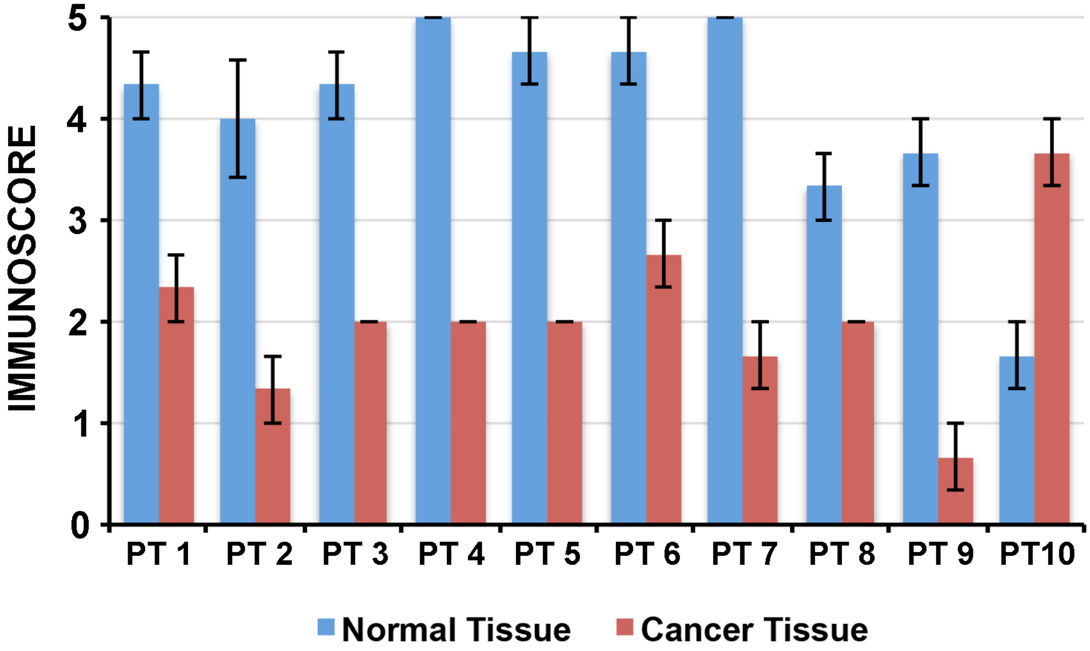

Supplementary Figure S5: GATA6 protein levels are down-regulated in colon cancer tissues. Bar graph representing the immunoscore index of GATA6 staining detected by immunohistochemistry. GATA6 positive cells were scored between 0 (no signal) and 5 (highest expression) in healthy tissues (blue) and tumor samples (red). Bars represent the mean percentage ± the SEM of three independent operators. PT= patient.
